# The role of BDNF in mediating the prophylactic effects of (R,S)-ketamine on fear generalization and extinction

**DOI:** 10.1038/s41398-022-02116-4

**Published:** 2022-08-25

**Authors:** James D. Ryan, Nathaniel Tse, Chienchun Huang, Ruirong Yang, Francis S. Lee

**Affiliations:** 1 Department of Psychiatry, Weill Cornell Medicine, New York, NY 10065 USA; 2 Feil Family Brain and Mind Research Institute, Weill Cornell Medicine, New York, NY 10065 USA; 3 Department of Pharmacology, Weill Cornell Medicine, New York, NY 10065 USA

**Keywords:** Hippocampus, Predictive markers

## Abstract

Fear generalization is a conserved survival mechanism that can become maladaptive in the face of traumatic situations, a feature central to certain anxiety disorders including posttraumatic stress disorder (PTSD). However, the neural circuitry and molecular mechanisms underlying fear generalization remain unclear. Recent studies have shown that prophylactic treatment with (R,S)-ketamine confers protective effects in stress-induced depressive behaviors and enhances contextual fear discrimination, but the extent to which these effects extend to fear generalization after auditory fear conditioning remains unclear. Here, we build on this work by using a behavioral model of fear generalization in mice involving foot shocks with differential intensity levels during auditory fear conditioning. We find that prophylactic (R,S)-ketamine treatment exerts protective effects that results in enhanced fear discrimination in wild type mice. As the growth factor, brain-derived neurotrophic factor (BDNF), has been shown to mediate the rapid antidepressant actions of (R,S)-ketamine, we used a loss-of-function BDNF mouse line (BDNF Val66Met) to determine whether BDNF is involved in (R,S)-ketamine’s prophylactic effects on fear generalization. We found that BDNF Val66Met mice were resistant to the protective effects of prophylactic (R,S)-ketamine administration on fear generalization and extinction. We then used fiber photometry to parse out underlying neural activity and found that in the ventral hippocampus there were significant fear generalization-dependent patterns of activity for wild type and BDNF Val66Met mice that were altered by prophylactic (R,S)-ketamine treatment. Overall, these findings indicate a role for the ventral hippocampus and BDNF signaling in modulating the mitigating effects of prophylactic (R,S)-ketamine treatment on generalized fear.

## Introduction

Posttraumatic stress disorder (PTSD) is a debilitating psychiatric illness that is characterized by exposure to a traumatic event and the subsequent development of a myriad of symptoms, including trauma re-experiencing (e.g., flashbacks or nightmares), hyperarousal, and avoidance behavior [1–3]. These symptoms proceed in part via the dysregulation of behaviors that normally serve adaptive functions [4–6]. One such behavior is fear generalization, an associative learning mechanism conserved across species in which fear acquired in relation to a conditioned stimulus or context (CS+) that consistently predicts the appearance of an aversive unconditioned stimulus (US) is transferred to another stimulus or context (CS−) not directly associated with the US [7, 8]. Trauma can induce maladaptive generalization to inappropriate and harmless stimuli [9, 10], and a tendency toward fear overgeneralization has notably been observed in PTSD patients [11, 12] though the underlying mechanisms remain unclear.

Recent work has begun to make significant progress in identifying the molecular and genetic mechanisms [13, 14] involved in PTSD and fear generalization behavior. Interestingly, human carriers of a single nucleotide polymorphism (SNP) in the brain-derived neurotrophic-factor (*BDNF)* gene that leads to a valine to a methionine substitution at codon 66 (BDNF Val66Met; rs6265) have increased anxiety-related behaviors [15, 16] as well as impairments in fear extinction learning [17]. Similarly, in a knock-in mouse line containing the variant BDNF, it has been previously shown that the Met allele is associated with decreased activity-dependent BDNF secretion from neurons in addition to increased anxiety-related behaviors [18] and impaired fear extinction [19]. Importantly, one study in humans investigating the impact of the BDNF Val66Met SNP uncovered a tendency towards increased fear generalization in BDNF Met carriers [20], but whether similar effects on fear generalization are present in BDNF Val66Met mice has yet to be explored.

The neural circuitry dysregulated in the development of fear generalization is speculated to include the basolateral amygdala, medial prefrontal cortex, and dorsal and ventral hippocampus [21–23]. In particular, one study identified neurons in the lateral amygdala that signaled generalized versus cue-specific associations, the distribution of which switched toward a greater proportion of generalizing neurons during the behavioral shift to fear generalization [24]. Furthermore, another study found that inhibiting long-term potentiation in the ventral hippocampus during fear learning was sufficient to increase fear generalization [25], suggesting that genotypes associated with deficits in synaptic plasticity (such as the BDNF Val66Met SNP [26]) may result in increased fear generalization behavior.

With regards to pharmacological treatment for PTSD, while serotonin reuptake inhibitors have been established to effectively reduce symptom severity in PTSD patients [27], they have substantial rates of non-response or suboptimal response [28]. In addition, among the treatments for PTSD, none provide protective effects before symptoms manifest. Interestingly, the N-methyl-D-aspartate (NMDA) receptor antagonist, (R,S)-ketamine, has been shown to reduce stress-induced behavioral and physiological changes when prophylactically administered prior to stress onset in rodents. Specifically, previous work has found that prophylactic (R,S)-ketamine administration blocks the induction of stress-induced depressive behavior [29, 30], while other work has indicated that (R,S)-ketamine administration prior to contextual fear conditioning results in a reduction in the expression of freezing behavior [31]. However, it is unclear whether this protective effect of (R,S)-ketamine administration extends to auditory fear conditioning paradigms, which are also more amenable to tracking real-time millisecond-level changes in neural activity and may be more informative with the increased temporal specificity offered by auditory tones. Additionally, while a role for the ventral hippocampus [32, 33] as well as other neural circuits or mechanisms that may mediate the protective prophylactic effects of (R,S)-ketamine administration have begun to be explored [34], the potential role of BDNF in mediating these effects remains unknown.

Here, we address these questions by first establishing a behavioral model of auditory fear generalization in wild type (WT) mice, and then demonstrate that a single prophylactic administration of (R,S)-ketamine is sufficient to enhance fear discrimination and subsequent fear extinction. Next, we assessed the role of BDNF in the fear generalization process by exposing BDNF^Met/Met^ mice to the strong shock fear generalization protocol with and without prophylactic (R,S)-ketamine administration. We demonstrate that prophylactic (R,S)-ketamine treatment enhances fear discrimination in WT mice but is less effective in BDNF^Met/Met^ mice. Finally, to parse out the neural circuits that mediate fear generalization, we used in vivo calcium imaging through fiber photometry recordings to measure population-level signaling activity in the ventral hippocampus (vCA1) during fear and safety memory recall and found robust, tone-evoked and genotype-dependent changes in activity in mice conditioned with strong foot shocks that is distinct from the weak foot shock conditions. Taken together, these findings identify the significant roles of the vCA1 and BDNF in mediating generalized fear and suggest the therapeutic utility of prophylactic (R,S)-ketamine in preventing fear generalization with applications for treating PTSD.

## Materials and methods

### Animals

Male adult (2–5 months of age) WT mice (C57BL/6N, Charles River Laboratories) and BDNF Val66Met (BDNF^Met/Met^) mice backcrossed onto a C57BL/6N background were used for all experiments. BDNF Val66Met mice were generated as described previously [18] and bred within the Weill Cornell Medicine animal colony. Animals were maintained on a 12-h light/dark cycle with ad libitum access to food and water. All behavioral testing was conducted during the 12-h dark cycle. All animal procedures were conducted following the rules of the Weill Cornell Medicine Institutional Animal Care and Use Committee and in accordance with the National Institutes of Health guide for the Care and Use of Laboratory Animals.

### Drugs

One week prior to the start of fear conditioning, mice were randomly assigned and received an intraperitoneal (i.p.) injection of either 0.9% NaCl or 30 mg/kg of (R,S)-ketamine (acquired from Weill Cornell Veterinary Services) with the (R,S)-ketamine dose and administration timing chosen based on previous prophylactic (R,S)-ketamine studies [29, 33].

### Cued fear conditioning and extinction

All fear acquisition and recall behavioral sessions occurred in sound-isolated boxes (Med Associates Inc., VT) equipped with white and infrared lights in addition to a tone generator. All sessions were video recorded and freezing behavior quantified using Video Freeze (Med Associates Inc., VT), and because all scoring was automated experimenters were not blinded to mouse treatment condition. Mice were exposed to two different contexts during the course of behavior, one for the initial fear conditioning (Context A, Day 1) and another context for the fear memory recall and extinction test days (Context B, Days 2 through 5). Contexts varied by lighting condition (white light on in Context A, off in Context B), floor and wall shape, and odor cues present (0.1% peppermint dissolved in 70% EtOH in Context A, 0.1% limonene dissolved in 70% EtOH in Context B). Chambers were cleaned with Quatricide (Pharmacal Research Laboratories, Waterbury CT) in between sessions. During the Day 1 habituation session in Context A, mice received five presentations each of two auditory cues (2.9 kHz or 12.5 kHz frequency, 10 s duration). Fear conditioning immediately followed the habituation session on Day 1, where one of the tones (CS+) became paired with and co-terminated with a 1-s duration electric foot shock (US; ten pairings with an average intertrial interval of 70 s but range of 40–100 s) whereas the other tone remained explicitly unpaired with the foot shock (CS−), for a total of ten randomly interleaved presentations each of the CS+ and CS−. Mice were randomly assigned to exclusively receive either weak (0.3 mA) or strong (1.0 mA) foot shocks. Foot shocks were delivered through a metal grid floor in the conditioning context (Context A). Twenty-four hours later (Day 2), mice were exposed to the fear recall context (Context B) and given five presentations each of the CS+ and CS− tones (10 s duration, with an average intertrial interval of 70 s) and their time spent freezing during the duration of the CS+ and CS− tones was quantified. To measure fear extinction, mice were then re-tested in Context B every 24 h (Day 3, 4, 5) for a total of 4 exposures using the same fear recall protocol (5 presentations each of 10-s CS+ and CS− tones), but with the order in which the tones were played randomized across days. The presentation order of CS+ and CS− tones was randomized throughout fear conditioning and fear extinction test sessions, such that a given CS+ or CS− tone would randomly be followed by either a CS+ or CS− tone.

## Results

**Fig. 1:**
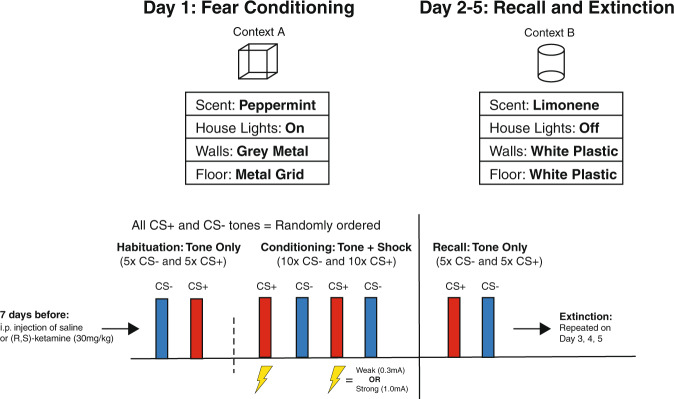
Schematic of fear conditioning and recall behavioral protocol. One week prior to testing, mice are injected with either saline or (R,S)-ketamine (30 mg/kg). Habituation and auditory fear conditioning on Day 1 in Context A, followed by four days of re-exposure to CS+ and CS− in absence of US (unconditioned stimulus, electric foot shock). Tone order (CS+ or CS−) randomly varied across testing days. Context A and Context B varied in terms of scent present in the chamber, lighting conditions, and chamber wall/floor material and dimensions.

In order to test the efficacy of prophylactic (R,S)-ketamine in reducing fear generalization behavior in mice, we first modified a fear generalization procedure established in rats [24]. WT mice were injected with either saline or (R,S)-ketamine (30 mg/kg) and then 1 week later were exposed to either a weak (0.3 mA) or strong shock (1.0 mA) auditory fear conditioning paradigm, followed by four additional days of fear memory recall testing in a novel context (Fig. 1).

### Weak shock conditioning promotes fear discrimination and is not altered by prophylactic (R,S)-ketamine treatment in WT mice

**Fig. 2:**
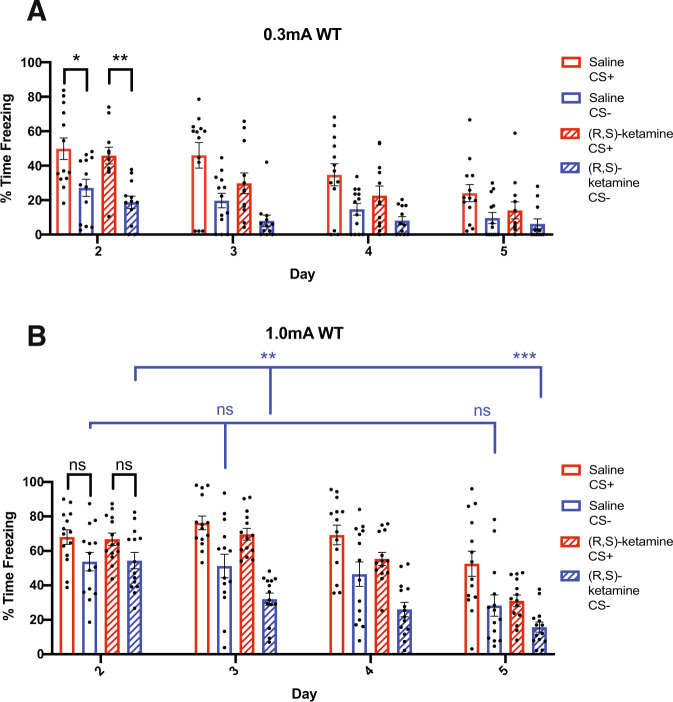
Auditory fear conditioning with a strong foot shock induces fear generalization behavior in wild type (WT) mice, which is reduced by prophylactic administration of (R,S)-ketamine. **A**, **B** Percent time freezing to CS+ and CS− tones following auditory fear conditioning with either weak (0.3 mA) or strong (1.0 mA) electric foot shocks. **A** Saline-injected WT mice (*N* = 13) and prophylactic (R,S)-ketamine-injected WT mice (*N* = 12) conditioned with weak shocks displayed significantly greater freezing behavior to CS+ tones compared to CS− tones on Day 2. **B** Saline-injected WT mice conditioned with strong shocks (*N* = 14) displayed similar levels of freezing behavior to both the CS+ and CS− tones during the Day 2 fear recall and did not show significant reductions in Day 2 v. Day 3 or Day 2 v. Day 5 freezing for the CS− tone. Prophylactic (R,S)-ketamine-injected WT mice conditioned with strong shocks (*N* = 14) froze at similar levels to the CS+ and CS− tones during the Day 2 fear recall session but exhibited a significant decrease in freezing to the CS− tone between the Day 2 session and Days 3 and 5 of testing. Error bars represent mean ± SEM. Three-way RM ANOVA with post hoc Tukey HSD tests, *** *p* < 0.001, **p < 0.01, *p < 0.05, ns = *p* > 0.05.

For the weak shock WT mice, we observed a significant main effect of Day (*F*_3,69_ = 32.36, *p* < 0.001) and Cue (*F*_1,23_ = 90.92, *p* < 0.001) on freezing, with an additional significant interaction effect of Day × Cue (*F*_3,69_ = 6.232, *p* = 0.0016). However, there was no main effect of Drug (*F*_1,23_ = 2.666, *p* = 0.116) and no interaction with Drug reached significance. We then performed post hoc Tukey HSD tests and found that saline-injected WT mice conditioned with a weak shock discriminated and froze significantly more in response to the CS+ cue than the CS− during the first fear recall session (Fig. 2A). Similarly, the prophylactic (R,S)-ketamine-injected WT mice conditioned with a weak shock exhibited fear discrimination and froze significantly more to the CS+ tone during the first fear recall test session (Fig. 2A). Additionally, the lack of a significant effect of drug treatment for the weak shock conditioned WT mice suggests that prophylactic (R,S)-ketamine does not significantly alter fear discrimination behavior in WT mice when conditioned with a weak foot shock.

### Strong shock conditioning induces fear generalization in WT mice which is rescued by prophylactic (R,S)-ketamine treatment

For the strong shock WT mice, we observed significant main effects of Day (*F*_3,78_ = 36.03, *p* < 0.001), Drug (*F*_1,26_ = 6.144, *p* = 0.0200), and Cue (*F*_1,26_ = 114.5, *p* < 0.001) on freezing, as well as significant interactions for Day × Drug (*F*_3,78_ = 3.427, *p* = 0.0211) and Day × Cue (*F*_3,78_ = 5.734, *p* = 0.0024). Follow-up analysis using post hoc Tukey HSD tests found that the saline-injected WT mice conditioned with a strong shock generalized and froze at similar levels to the CS+ and CS− cues during the first fear recall test session (Fig. 2B). This result differed from that observed in the saline-injected WT mice conditioned with a weak shock, which displayed fear discrimination and froze significantly more to the CS+ tone than the CS− tone during the first fear recall test session, indicating that this fear conditioning procedure can induce fear generalization behavior in WT mice in a shock intensity-dependent manner. Similar to the saline-injected WT mice, the prophylactic (R,S)-ketamine-injected WT mice also generalized to the CS+ and CS− tones during the first fear recall test session (Fig. 2B), indicating that prophylactic (R,S)-ketamine does not impact early fear discrimination behavior in our paradigm. However, we did observe a significant main effect of drug treatment for the strong shock-conditioned WT animals, and post hoc tests revealed that the prophylactic (R,S)-ketamine-injected WT mice demonstrated a significant reduction in freezing to the CS− tone between Day 2 and Day 3, and between Day 2 and Day 5 (Fig. 2B). Conversely, the saline-injected WT mice conditioned with a strong shock did not show a significant change in freezing in response to the CS− tone between the Day 2 and Day 3 fear recall test sessions, and even between Day 2 and Day 5 there was no significant change in CS− freezing (Fig. 2B). These results suggest that while prophylactic (R,S)-ketamine treatment does not affect fear generalization behavior during early test recall sessions, it does ultimately enhance fear discrimination by promoting fear extinction for the CS− cue during later test sessions. The finding that prophylactic (R,S)-ketamine produced a protective effect on fear generalization in the strong shock conditioned WT mice (but not in the weak shock conditioned mice) is also consistent with findings from studies investigating the antidepressant properties of (R,S)-ketamine, where (R,S)-ketamine was found to produce robust antidepressant effects in animals exposed to high-stress situations that were absent or reduced in animals exposed to either weak stressors or no stress [35, 36]. This suggests that the protective effects of prophylactic (R,S)-ketamine treatment on fear discrimination may be dependent on exposure to a particularly stressful conditioning experience, and do not manifest if the intensity of the stressor is reduced.

### BDNF^Met/Met^ mice conditioned with a weak shock demonstrate increased fear generalization which is not altered by prophylactic (R,S)-ketamine treatment

As (R,S)-ketamine’s antidepressant effects are in part mediated by BDNF [37], we examined whether (R,S)-ketamine’s prophylactic effects on fear generalization and fear extinction are BDNF-dependent. Utilizing the same fear conditioning paradigm previously described, BDNF^Met/Met^ mice were injected with saline or (R,S)-ketamine (30 mg/kg) 1 week before auditory fear conditioning.

**Fig. 3:**
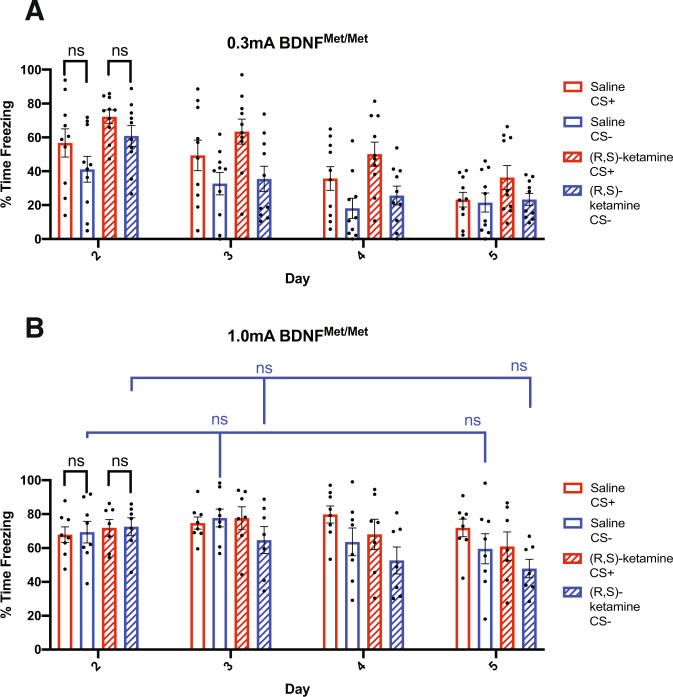
The protective effects of prophylactic (R,S)-ketamine administration on fear generalization are impaired in BDNF^Met/Met^ mice. **A** BDNF^Met/Met^ mice injected with saline and conditioned with weak shocks (*N* = 10) generalize and display similar levels of freezing behavior in response to the CS+ and CS− tones during the Day 2 fear recall test session, and prophylactic (R,S)-ketamine administration prior to weak shock conditioning does not significantly alter fear discrimination in BDNF^Met/Met^ mice (*N* = 10). **B** BDNF^Met/Met^ mice injected with saline and conditioned with strong shocks (*N* = 8) generalize and freeze at similar levels to the CS+ and CS− tones during the Day 2 fear recall test session and do not show reductions in freezing to the CS− tone across testing. BDNF^Met/Met^ mice injected with prophylactic (R,S)-ketamine and conditioned with strong shocks (*N* = 7) freeze at similar levels to the CS+ and CS− tones during the Day 2 fear recall test session and do not show reduced freezing to the CS− tone across the other testing days. Error bars represent mean ± SEM. Three-way RM ANOVA with post hoc Tukey HSD tests, ns = *p* > 0.05.

For the weak shock BDNF^Met/Met^ mice, we observed significant main effects of Day (*F*_3,54_ = 22.05, *p* < 0.001) and Cue (*F*_1,18_ = 76.65, *p* < 0.001) on freezing, but not Drug (*F*_1,18_ = 2.741, *p* = 0.1151). Additionally, we observed a significant interaction between Day × Cue (*F*_3,54_ = 3.123, *p* = 0.0413) but no interaction with Drug reached significance. Interestingly, using post hoc Tukey HSD tests we found that the weak shock saline-injected BDNF^Met/Met^ mice generalized and froze at similar levels to the CS+ and CS− tones during the Day 2 fear recall test session (Fig. 3A), differing from the weak shock saline-injected WT mice that discriminated and froze significantly more to the CS+ tone when tested at the same time point. This early fear generalization effect was also observed in the prophylactic (R,S)-ketamine injected BDNF^Met/Met^ mice, which likewise also generalized and froze at similar levels to the CS+ and CS− tones during the Day 2 fear recall test session (Fig. 3A). These results suggest that fear conditioning with a weak shock is sufficient to induce fear generalization behavior in BDNF^Met/Met^ mice but not WT mice, which is consistent with previous research indicating greater fear generalization in human BDNF Val66Met SNP carriers [20]. Additionally, the lack of fear discrimination exhibited by the prophylactic (R,S)-ketamine injected BDNF^Met/Met^ mice indicates that prophylactic (R,S)-ketamine does not affect fear discrimination behavior in BDNF^Met/Met^ mice under weak shock conditions.

### Prophylactic ketamine treatment does not rescue fear generalization induced by strong shock conditioning in BDNF^Met/Met^ mice

For the strong shock BDNF^Met/Met^ mice, we observed a significant main effect of Day (*F*_3,39_ = 5.194, *p* = 0.0057) and Cue (*F*_1,13_ = 17.12, *p* = 0.0012) but not Drug (*F*_1,13_ = 0.6787, *p* = 0.4249). We also observed a significant interaction between Day × Cue (*F*_3,39_ = 4.597, *p* = 0.0156) but did not observe any Drug interactions with Day or Cue. Follow-up post hoc Tukey HSD tests indicated that strong shock saline-injected BDNF^Met/Met^ mice generalized and froze at similar levels to the CS+ and CS− tones during the Day 2 fear recall test session, similar to the strong shock-induced generalization effect observed in the saline-injected WT mice, and that likewise the prophylactic (R,S)-ketamine injected BDNF^Met/Met^ mice also demonstrated fear generalization during the Day 2 fear recall test session (Fig. 3B). The saline-injected BDNF^Met/Met^ mice conditioned with a strong shock also did not show significant reductions in freezing to the CS− tone over the course of the testing days (Fig. 3B), similar to the effects seen in the strong shock-conditioned saline-injected WT mice. However, unlike the strong shock-conditioned prophylactic (R,S)-ketamine injected WT mice that demonstrated significant reductions in freezing to the CS− tone from Day 2 to Day 3 and Day 5, the prophylactic (R,S)-ketamine injected BDNF^Met/Met^ mice did not show significant reductions in CS− freezing over the course of testing (Fig. 3B). Together, these results indicate that the rescuing effect of prophylactic (R,S)-ketamine treatment on fear generalization under strong shock conditions is impaired in the BDNF^Met/Met^ mice, which is consistent with findings from the antidepressant literature that (R,S)-ketamine is also less effective at exerting its antidepressant effects in BDNF^Met/Met^ animals that exhibit a reduction in activity-dependent BDNF secretion [38].

### Prophylactic (R,S)-ketamine alters ventral hippocampal activity during fear generalization for salient cues

**Fig. 4:**
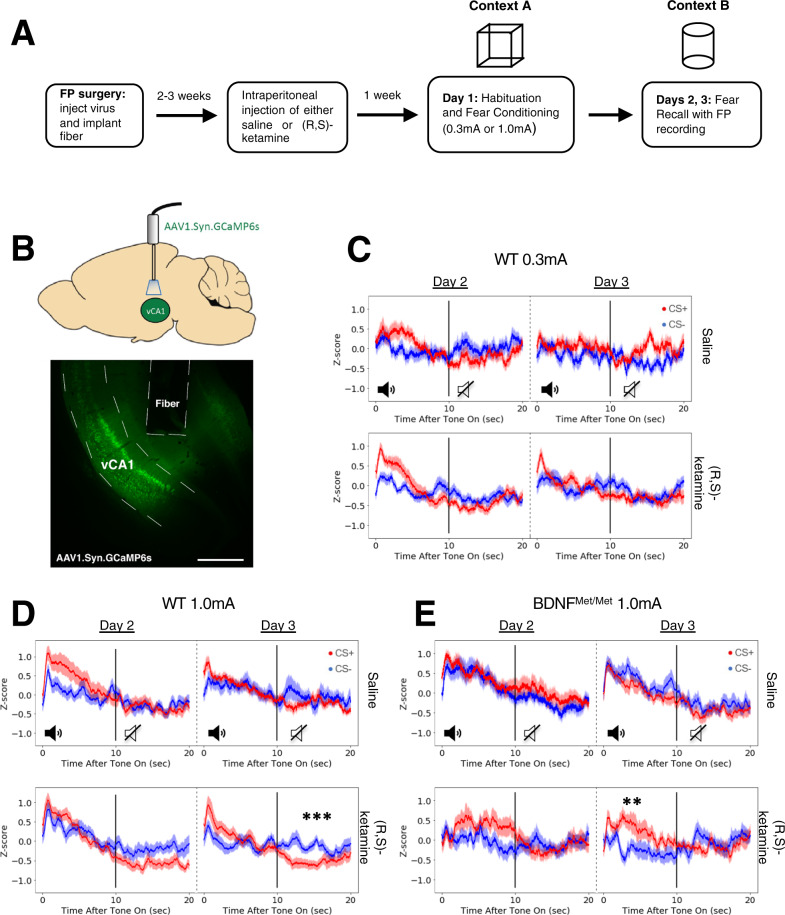
Fiber photometry reveals prophylactic (R,S)-ketamine enhances differential vCA1 activity immediately following cue presentation. **A** Schematic detailing timeline of surgery, drug administration, fear conditioning and fiber photometry recall tests. **B** Diagram showing viral delivery of GCaMP6s to vCA1 cells, with representative image of an optic fiber implanted above delivery site (scale bar = 500 µm). **C**–**E** Average fluorescent signal across 10 s of tone presentation, followed by 10 s post-tone interval (red denotes signal during CS+, blue denotes signal during CS−, vertical line denotes time of tone off). Averaged traces displayed for test Days 2 and 3. Signal from saline-injected animals given on top, signal from prophylactic (R,S)-ketamine-injected animals given on bottom. **C** Following fear conditioning at weak (0.3 mA) shock in WT mice, saline (*N* = 6) and (R,S)-ketamine-administered (*N* = 7) groups did not display significantly different signal between CS+ and CS− presentations on either day, either during tone presentation or immediately after. **D** On both test days following fear conditioning at 1.0 mA shock, average fluorescent signal from the WT saline-administered group (*N* = 6) did not differ between CS+ and CS− presentations, either during tone presentation or immediately after. However, average signal in the post-tone period was significantly higher following CS− presentation compared to CS+ in the prophylactic (R,S)-ketamine-administered group (*N* = 6), on Day 3 following strong shock conditioning. **E** BDNF^Met/Met^ mice conditioned with 1.0 mA shocks did not display any difference in signal in the post-tone period, regardless of drug treatment or test day (*N* = 6 saline/*N* = 5 ketamine); however, (R,S)-ketamine-injected BDNF^Met/Met^ mice displayed greater CS+ activity during cue presentation on Day 3. Paired, two-tailed *t* tests with Bonferroni corrections for multiple comparisons, ***p < 0.001, **p < 0.01.

Given the evidence for the role of the ventral hippocampus in regulating fear generalization [8], we next used fiber photometry to quantify population-level neuronal activity in the vCA1 both during and immediately following presentation of the CS+ and CS− cues. Mice received unilateral stereotaxic injections of an AAV1 encoding the calcium indicator GCaMP6s in the vCA1, and an optical fiber was implanted above the vCA1 to record cellular activity (Fig. 4A). Two to three weeks following surgery, mice were injected either with saline or (R,S)-ketamine (30 mg/kg). One week after injection, mice were subjected to the same fear conditioning paradigm (Fig. 1A), but on Days 2-3, the time frame in which (R,S)-ketamine-administered mice began to display fear discrimination behavior, activity in the vCA1 was recorded via a fiber optic patch-cord. Following behavioral testing, virus injection and fiber placement in the vCA1 was verified and only animals with appropriate placement were analyzed.

We previously demonstrated that WT mice receiving strong conditioning shocks generalized and froze to both the CS+ and CS− cues, and that prophylactic administration of (R,S)-ketamine reduced this generalization effect beginning on test Day 3. Therefore, we investigated whether CS− and CS+ cue presentations would elicit in saline and (R,S)-ketamine-administered mice patterns of vCA1 activity in line with generalizing and discriminating behavior. In the strong shock group, neither saline nor (R,S)-ketamine-injected WT animals displayed any difference in vCA1 activity during the 10 s the CS+ tone or CS− tone was on during Day 2, with a similar lack of an effect during tone presentation for the CS+ tone and CS− tone on Day 3 (Fig. 4D, top). However, on Day 3, (R,S)-ketamine-injected WT animals showed significantly greater vCA1 activity in the 10 s following the end of CS− presentation compared to the 10 s period following CS+ presentation (Fig. 4D, bottom). These findings indicate that (R,S)-ketamine’s enhancement of fear discrimination occurs via modulation of the CS− cue offset response.

In the weak shock group we found no difference in vCA1 activity during or immediately following CS+ or CS− cue presentations in both saline and (R,S)-ketamine-administered animals on either Day 2 or 3 (Fig. 4C). Although we had expected vCA1 activity in weak shock mice to differ in response to the two cue types, reflecting their discriminating freezing behavior, these weak shock vCA1 activity data suggest that the ability to discriminate between CS+ and CS− cues does not necessitate differentiated cue offset responses; it may be the case then that post-tone vCA1 activity reflects an updating of CS+ and CS− cue memory. Magnitude of post-tone activity may therefore depend on the salience of the original memory. As such, the memory of a CS− cue in a discriminating mouse may not undergo significant updating over extinction sessions, matching the post-tone activity we observed in weak shock WT mice.

### Prophylactic (R,S)-ketamine fails to differentially modify post-cue ventral hippocampal activity in BDNF^Met/Met^ mice

Building off our behavioral findings that BDNF^Met/Met^ mice are resistant to the protective effects of prophylactic (R,S)-ketamine treatment, we next investigated whether vCA1 activity in BDNF^Met/Met^ mice would reflect their unique behavioral phenotype and resistance to the effects of (R,S)-ketamine. In line with our hypothesis that post-tone vCA1 activity represents the updating of fear memory, we found that on both test days, saline-injected strong shock conditioned BDNF^Met/Met^ mice showed no difference in vCA1 activity during or immediately following CS+ and CS− cues (Fig. 4E, top). Furthermore, we found that (R,S)-ketamine administered BDNF^Met/Met^ mice showed no difference in vCA1 activity in the CS+ and CS− post-cue period on either Day 2 or Day 3 (Fig. 4E, bottom). The during-cue vCA1 activity in these (R,S)-ketamine administered mice also did not differ significantly between CS+ and CS− cues on Day 2. However, on Day 3, vCA1 activity was significantly greater during CS+ tone presentation compared to during CS− tone presentation (Fig. 4E, bottom). This differential vCA1 response does not have an obvious behavioral link, given that (R,S)-ketamine administered strong shock BDNF^Met/Met^ mice do not display discriminating freezing (Fig. 3B). Instead, this during-cue vCA1 activity difference may indicate that while (R,S)-ketamine’s behavioral effects are abolished in a low-BDNF environment, (R,S)-ketamine is still able to exert some sub-threshold effects on fear discrimination circuitry including the vCA1. Taken together with our WT mice results, these data provide evidence that prophylactic (R,S)-ketamine may modulate vCA1 activity in a BDNF-dependent manner to enhance fear discrimination.

## Discussion

Here, we have shown that prophylactic administration of (R,S)-ketamine one week prior to fear conditioning with strong foot shocks enhances fear discrimination and fear extinction in WT mice. We also find that the protective effects of prophylactic (R,S)-ketamine administration are reduced in BDNF Val66Met mice, which have a deficit in activity-dependent BDNF secretion, suggesting BDNF signaling as a mechanism by which prophylactic (R,S)-ketamine treatment enhances fear discrimination and extinction. Further, using in vivo calcium imaging in freely moving animals during the fear recall and extinction test sessions, we also identify shock intensity and genotype-dependent effects of prophylactic (R,S)-ketamine in the vCA1 region of the hippocampus that are associated with the behavioral effects of prophylactic (R,S)-ketamine on fear generalization and fear extinction.

The current findings suggesting a role for BDNF in mediating the prophylactic effects of (R,S)-ketamine are consistent with previous work on the role of BDNF in mediating the antidepressant effects of (R,S)-ketamine [39]. Of particular interest, recent work has found that the antidepressant effects of (R,S)-ketamine are significantly impaired in BDNF Val66Met mice [38]. The proposed mechanism is that the BDNF Val66Met SNP confers constitutive basal synaptic deficits that show no added benefit from the synaptogenic effects of (R,S)-ketamine, which ultimately prevents (R,S)-ketamine from producing antidepressant effects. This finding is particularly interesting in relation to a later study that found there is a similar resistance to the antidepressant effects of (R,S)-ketamine in patients with depression who carry the Met allele [40]. These previous studies inform the current work, where we demonstrate that BDNF Val66Met mice are also less responsive to the protective effects of prophylactic (R,S)-ketamine and highlight a potential mechanism of action by which this effect occurs. One possibility is that prophylactic (R,S)-ketamine treatment may induce BDNF-dependent constitutive changes that affect basal synaptic plasticity in a way that promotes increased fear discrimination and enhanced extinction in WT mice which is diminished in the BDNF^Met/Met^ mice. However, elucidating the precise molecular mechanism by which the BDNF Val66Met polymorphism impairs the response to prophylactic (R,S)-ketamine remains an active area of inquiry.

Importantly, one study [41] using a similar shock intensity-induced fear generalization protocol that was used in our work and that of others [24] found that (R,S)-ketamine attenuated fear generalization in mice when administered 22 h after fear conditioning. Conversely, the same study found that prophylactic administration of (R,S)-ketamine prior to fear conditioning using a similar to that regimen used in the current study (30 mg/kg i.p., one week before) failed to prevent fear generalization. In this previous study, the authors analyzed only one day of fear recall occurring 24 h after conditioning, at which point we similarly did not see effects of prophylactic (R,S)-ketamine treatment on fear generalization behavior. However, in our strong intensity shocked WT mice that received prophylactic (R,S)-ketamine, we observed a marked and persistent increase in fear discrimination present by Day 3, suggesting that multiple days of fear extinction training facilitate emergence of the protective effects of prophylactic (R,S)-ketamine treatment. In addition, in this previous study [41] it was also shown that in their control conditions, BDNF levels decreased significantly in the BLA 2 h after fear conditioning, which contrasts with prior studies demonstrating increased BDNF levels after fear conditioning [42, 43]. Of note, in both our study and the previously referenced study only male animals were used. Other work using prophylactic (R,S)-ketamine prior to chronic stress exposure found that (R,S)-ketamine treatment exerted a protective effect by preventing the onset of chronic stress-induced behaviors in both male and female mice [44], but that the prophylactic efficacy of (R,S)-ketamine in females is absent in ovariectomized females, suggesting a role for ovarian hormones in mediating the efficacy of prophylactic (R,S)-ketamine. Thus, it would be informative in future work to determine the extent to which the prophylactic effects of (R,S)-ketamine on fear generalization and extinction are hormone-dependent and modulated by sex differences.

An additional area of investigation for this study was to elucidate the neural circuitry important for exerting the protective effects of prophylactic (R,S)-ketamine administration. One recent study [33] demonstrated that prophylactic (R,S)-ketamine administration increased ΔFosB expression in the ventral, but not dorsal, hippocampus of stressed mice and that transcriptional silencing of ΔFosB in the ventral CA3 region of the hippocampus (but not the dentate gyrus) blocked the attenuating effects of prophylactic (R,S)-ketamine on learned contextual fear, which was reversed by viral overexpression of ΔFosB in the ventral CA3. While these results convincingly demonstrate a role for both ΔFosB and the ventral hippocampus in mediating the protective effects of prophylactic (R,S)-ketamine, they leave open the extent to which prophylactic (R,S)-ketamine alters fear-associated ventral hippocampal activity in freely moving mice, an area which is informed by the current study.

Recent work using fiber photometry to record from dopaminergic neurons in the ventral tegmental area during fear extinction found the presence of a robust post-cue prediction-error like signal associated with extinction learning [45], with similar work further supporting that neural activity elicited by tone onset may represent processing related to the salience of the cue while activity elicited by tone offset represents predication error-related processing that contributes to fear extinction learning [46]. Taking these findings into account with our present data, we hypothesize that differences in tone offset elicited activity between groups may reflect the progressive learning of a novel extinction memory or updating of an existing fear memory. Our finding that strong intensity shocked WT mice given prophylactic (R,S)-ketamine demonstrated a robust tone offset response to the CS− cue that was absent in their saline-injected counterparts suggests that the protective effects of prophylactic (R,S)-ketamine treatment on fear generalization are associated with enhanced discrimination for the CS− cue mediated by the vCA1. The lack of such an effect in the strong intensity shocked BDNF^Met/Met^ mice, regardless of whether they received prophylactic (R,S)-ketamine or saline treatment, was also associated with the reduced ability of prophylactic (R,S)-ketamine treatment to enhance fear discrimination or extinction in the BDNF^Met/Met^ mice. Surprisingly, the sole instance in which we observed a significant difference between averaged CS+ and CS− tone onset elicited activity occurred during the Day 3 test session for the prophylactic (R,S)-ketamine-injected BDNF^Met/Met^ mice, an effect not clearly associated with any of the prior behavioral data from prophylactic (R,S)-ketamine-injected BDNF^Met/Met^ mice, suggesting there is a tighter linkage between alterations in behavior and neural activity induced by prophylactic (R,S)-ketamine treatment that manifests during tone offset rather than onset in the ventral CA1. One limitation inherent to our fiber photometry data is that commonly-used anesthetics may induce long-lasting changes in fear learning and stress-induced behaviors, which has been observed with both isoflurane [47] and the ketamine/xylazine [48] cocktails similar to that used in the present study, leaving open the possibility that the experience of undergoing surgery and receiving anesthetic might themselves have influenced behavior in our fiber photometry mouse cohorts. Additionally, in the present study our fiber photometry recordings indicated broad ventral CA1 activity and did not selectively isolate activity between the ventral CA1 and other regions involved in fear recall and extinction. Notably, recent work using fiber photometry during learned safety recall has indicated differences in neural activity elicited by cues associated with threat or safety tones in ventral CA1 neurons dependent on whether they project to the prelimbic or infralimbic portions of the medial prefrontal cortex [49]. Thus, our ability to parse the relationship between ventral CA1 activity and fear recall and extinction may be confounded by measuring the totality of ventral hippocampal activity. An additional limitation is that by using a synapsin-driven GCaMP6s virus we infected all neuron subtypes within the hippocampus, including both excitatory and inhibitory cells, and thus our fiber photometry signal represents the concerted activity of vCA1 neurons as a whole but does not indicate the activity of particular neuron subtypes. It would be invaluable for future work to selectively isolate neural subtype activity in the vCA1 and identify the differing relevance of distinct subtypes in mediating the patterns of tone-evoked activity observed in this study.

In sum, the present work demonstrates that prophylactic (R,S)-ketamine administration enhances fear discrimination and fear extinction learning in a manner that is dependent on BDNF signaling and is associated with learning-induced changes in ventral hippocampal activity. Further, the identification of a human BDNF SNP that is resistant to the protective effects of prophylactic (R,S)-ketamine may also inform future PTSD-related clinical studies aimed at assessing the efficacy of prophylactic (R,S)-ketamine treatment based on the patient’s unique genetic background.

## Supplementary information

Supplementary Methods
