# Supplementary material for: The role of BDNF in mediating the prophylactic effects of (R,S)-ketamine on fear generalization and extinction
Source: Transl Psychiatry. 2022 Aug 25;12:346. doi: 10.1038/s41398-022-02116-4 (PMC9411535; doi:10.1038/s41398-022-02116-4)
Supplement: Supplementary file 1 — Supplementary Methods [file 41398_2022_2116_MOESM1_ESM.pdf]

Supplementary Materials and Methods

Fiber Photometry: Fiber photometry surgeries were performed three to four weeks prior to behavioral experiments. Mice were anesthetized with a (R,S)-ketamine/xylazine cocktail (100 mg/kg (R,S)-ketamine and 10 mg/kg xylazine) and then placed on a stereotactic frame. A unilateral hole was drilled over the vCA1 (A/P = -3.2, M/L = ±3.1) and a 10 µL Nanofil syringe (World Precision Instruments) fitted with a 33-gauge flat needle and connected to an infusion pump was used to microinject 150 nL of AAV1.Syn.GCaMP6s.WPRE.SV40 (Penn Vector Core) into the vCA1 (D/V = -3.2) at a rate of 50 nl/min. Next, a 400 µm diameter optical fiber (Doric) was then implanted into the vCA1 and secured to the skull with Metabond (Parkell). For recording GCaMP6s-induced fluorescent signal during fear memory recall and extinction a custom-built fiber photometry rig based on a previously described design[1,2] was used. Briefly, to induce GCaMP6s fluorescence a 470nm wavelength light emitted at 521 Hz from an LED (Thorlabs) was passed through a filter (Semrock, FF02-472/30) and then connected to the fiber implant via 0.48 NA fiber optic patch cord (Doric). GCaMP6s-induced fluorescent signal was then transmitted through the fiber optic patch cord, separated from the excitation light with a dichroic (Semrock, FF495-Di03), passed through a single band filter (Semrock, FF01-535/50), and finally focused on a photodetector (Newport, Model 2151) and recorded by a real-time processor (Tucker Davis Technologies). In order to time lock fluorescent signal with tone presentations, a TTL pulse was sent from the behavioral set-up to the fiber photometry rig that precisely coincided with the beginning and the end of the test session.

Fiber Photometry Data Analysis: Raw fluorescent signal data was imported and analyzed using custom MATLAB (MathWorks) software and Python scripts. A dynamic fluorescent baseline value was acquired by identifying the median value during a rolling window of 80 seconds (40

seconds before and 40 seconds after) around a given data point. To calculate dF/F, this baseline value was then subtracted from every given data point across the entire session and every outcome then divided by the same median. To control for inter-individual variability, calculated dF/F was z-scored for all mice and these z-scored dF/F values ultimately used for analysis. Average fluorescent activity was calculated during 10 second periods coinciding with when the CS+ or CS- tones came on (tone onset) and the following 10 seconds when the CS+ or CS- tones turned off (tone offset).

Histology: To confirm viral expression and fiber placement, after the conclusion of behavioral experiments mice were deeply anesthetized with Euthasol (0.1 mL/10g body weight) and transcardially perfused with 30ml 0.9% NaCl followed by 120 ml of 4% paraformaldehyde in 0.1M phosphate buffer. Brains were then dissected out and post-fixed in 4% paraformaldehyde in 0.1M phosphate buffer at 4°C for 12-24 hours and then transferred to a 30% sucrose solution at 4°C for 48-96 hours until brains sank in the solution. Forty micrometer-thick coronal sections were then cut on a freezing microtome and mounted on microscope slides for analysis under a fluorescent microscope (Nikon Eclipse 80i) using Stereo Investigator software (MBF Bioscience). Mice with either viral expression of GCaMP6s that was not localized to the vCA1 or had fiber optic implants not correctly placed over the vCA1 were excluded from analysis.

Statistical analyses: Sample size was chosen based on a previously published protocol[3] and using power tests (power = 0.8). Three-way repeated measures ANOVAs with *post hoc* Tukey tests following significant main effects were used to compare freezing behavior within each successive testing day between drug treatments (saline and prophylactic (R,S)-ketamine). Fiber photometry data was analyzed using two-tailed paired t-tests between CS+ and CS- activity averaged across 10-second bins (tone onset and tone offset). All fiber photometry p-values were Bonferroni

corrected for multiple comparisons. No animals were excluded based on abnormal freezing behavior. One animal (a BDNF Val66Met mouse injected with saline and conditioned with the 0.3mA shock) was euthanized prior to the Day 3 recall test session due to bite wounds and was thenceforth excluded from analysis. All statistical analyses were performed using Prism software, and the critical value for significance was set at an alpha level of 0.05. All data are presented as mean ± SEM.

Code availability: All custom MATLAB and Python code used for data processing and analysis is available upon reasonable request.

References

1 Gunaydin, L. A. *et al.* Natural neural projection dynamics underlying social behavior. *Cell* **157**, 1535-1551 (2014).

2 Marcus, D. J. *et al.* Endocannabinoid Signaling Collapse Mediates Stress-Induced Amygdalo-Cortical Strengthening. *Neuron* (2020).

3 Ghosh, S. & Chattarji, S. Neuronal encoding of the switch from specific to generalized fear. *Nat Neurosci* **18**, 112-120, doi:10.1038/nn.3888 (2015).
